# Total Aglycones from *Marsdenia tenacissima* Increases Antitumor Efficacy of Paclitaxel in Nude Mice

**DOI:** 10.3390/molecules190913965

**Published:** 2014-09-05

**Authors:** Rui-Jing Zhu, Xiao-Ling Shen, Ling-Lin Dai, Xiang-Ying Ai, Ru-Hua Tian, Rong Tang, Ying-Jie Hu

**Affiliations:** Laboratory of Chinese Herbal Drug Discovery, Tropical Medicine Institute, Guangzhou University of Chinese Medicine, Guangzhou 510405, China

**Keywords:** *Marsdeniae tenacissimae* Caulis, total aglycones, polyoxpregnane, paclitaxel, HeLa, KB-3-1, chemosensitizer

## Abstract

*Marsdeniae tenacissimae* Caulis (MTC) is a Chinese herbal medicine used mainly for treatment of cancer, whose pharmacologically active constituents responsible for its *in vivo* activity and clinical efficacy have not been clearly elucidated. In this study, total aglycones of MTC (ETA) showed the ability to sensitize KB-3-1, HeLa, HepG2 and K562 cells to paclitaxel treatment. More inspiringly, ETA markedly enhanced the antitumor activity of paclitaxel in nude mice bearing HeLa or KB-3-1 xenografts. Compared to treatment with paclitaxel alone, treatment with combination of paclitaxel and ETA achieved significant reduction in volume and weight of HeLa tumors (*p* < 0.05), and remarkable inhibition to the growth of KB-3-1 tumors (*p* < 10^−6^). ETA was characterized by the presence of a group of tenacigenin B ester derivatives, among which four reference compounds, 11α-*O*-tigloyl-12β-*O*-acetyltenacigenin B, 11α,12β-di-*O*-tigloyltenacigenin B, 11α-*O*-2-methylbutanoyl-12β-*O*-tigloyltenacigenin B, and 11α-*O*-(2-methylbutanoyl)-12β-*O*-benzoyltenacigenin B, accounted for 42.14% of the total peak area of 19 detectable components assayed by HPLC. Our study has identified ETA as a promising sensitizer for cancer chemotherapy.

## 1. Introduction

Cancer is one of the major diseases in China. In 2009, there were 3.12 million new cases diagnosed with cancer, and up to 2.7 million patients died of cancer. Surgery, radiation and drugs are effective means for treatment of cancer, but the average five-year survival rate of cancer patients is only 20.0%–30.0% [1]. A main cause for the high cancer mortality is drug resistance. Tumors have either intrinsic or acquired resistance to a number of anticancer agents, leading to therapeutic failure [2]. Natural products of plant origin play very important roles in research and development of cancer therapeutics [3]. For example, a number of clinically used anticancer drugs are of plant origin, including the Vinca alkaloids, taxanes, podophyllotoxins, and camptothecins [4,5,6]. *Marsdeniae tenacissimae* Caulis [origin: dried stems of the Asclepiadaceous plant *Marsdenia tenacissima* (Roxb.) Wight et Arn] is a Chinese herbal medicine used for the treatment of coughs, rheumatism, carbuncles and tumors [7]. A water soluble extract of *M. tenacissimae* (MTE) is used as raw material for Xiao-Ai-Ping injection (XAP), a Chinese herbal drug approved for the treatment of cancer [8]. If co-administered with anticancer agents such as platinum drugs XAP is reported to be able to enhance the clinical effects of chemotherapy in the treatment of lung cancer, liver cancer or gastric cancer, *etc.* [9,10]. Pharmacological studies showed that XAP or its chloroform extract may increase gefitinib sensitivity in drug resistant non-small cell lung cancer cells [11], sensitize MG63 cells to doxorubicin-induced apoptosis [12], induce apoptosis of hematologic neoplasm cells [13], or affect angiogenesis [14]. So far, more than 40 pregnane derivatives were identified from *M. tenacissima* [15], and several of them were reported to be cytotoxic to cancer cell lines [16], or capable of reversing multidrug resistance in Pgp overexpressing HepG2 cells [17]. Though the pharmacologically active constituents in MTC have not been clearly elucidated yet, liposoluble pregnane derivatives are thought to be responsible or partially responsible for its *in vivo* anticancer or chemosensitizing activities [11,12,13,14,15,16,17,18]. In this study, a hydrophobic extract of total pregnane aglycones (ETA) was obtained from the hydrolytic product of the crude glycosides of MTC. The possibility for clinical usage of ETA in combination with chemotherapy for treatment of human cancers, *in vitro* and *in vivo* activity of ETA in increasing antitumor effect of paclitaxel (taxol), as well as a quality control method for ETA were investigated*.*

## 2. Results and Discussion

### 2.1. Enhanced Inhibitory Effect of Paclitaxel on Cell Viability by ETA

In the human hepatoma cell line HepG2, leukemia cell line K562, oral epidermoid carcinoma cell line KB-3-1 and human cervical cancer cell line HeLa, cell viabilities in the presence of 10 μg/mL of ETA were over 98%, thus 10 μg/mL was chosen as the working concentration of ETA in investigations of the ability of ETA to enhance the *in vitro* anticancer activity of paclitaxel. Paclitaxel concentrations that inhibited 50% of cell viability (IC_50_) in the absence or presence of 10 μg/mL ETA in K562, HeLa, KB-3-1 and HepG2 cell lines were measured and compared. As shown in Table 1, addition of 10 μg/mL of ETA significantly decreased the IC_50_ values of paclitaxel in HeLa, HepG2 and KB-3-1 cells, implying that the anticancer activity of paclitaxel in the three cell lines was enhanced by ETA.

**Table 1:** IC_50_ values of paclitaxel in human cancer cell lines (ng/mL). Cancer cells were treated with various concentrations of paclitaxel alone (Control) or in the presence of 10 μg/mL of ETA for 48 h. Cell viability was measured by CCK-8 assay. Data were expressed as Mean ± SD of three independent experiments. * *p* < 0.05 and ** *p* < 0.01 compared to control.

Treatment	Tumor Cell Lines			
	K562	HeLa	HepG2	KB-3-1
Paclitaxel alone (Control)	4.8 ± 0.4	17.1 ± 4.1	67.2 ± 12.3	8.1 ± 1.3
Paclitaxel + 10 μg/mL ETA	3.8 ± 0.6	8.3 ± 1.0 *	14.1 ± 8.5 **	5.1 ± 0.6 *

### 2.2. Enhanced Inhibitory Effect of Paclitaxel on Tumor Growth in Nude Mice by ETA

**Figure 1:**
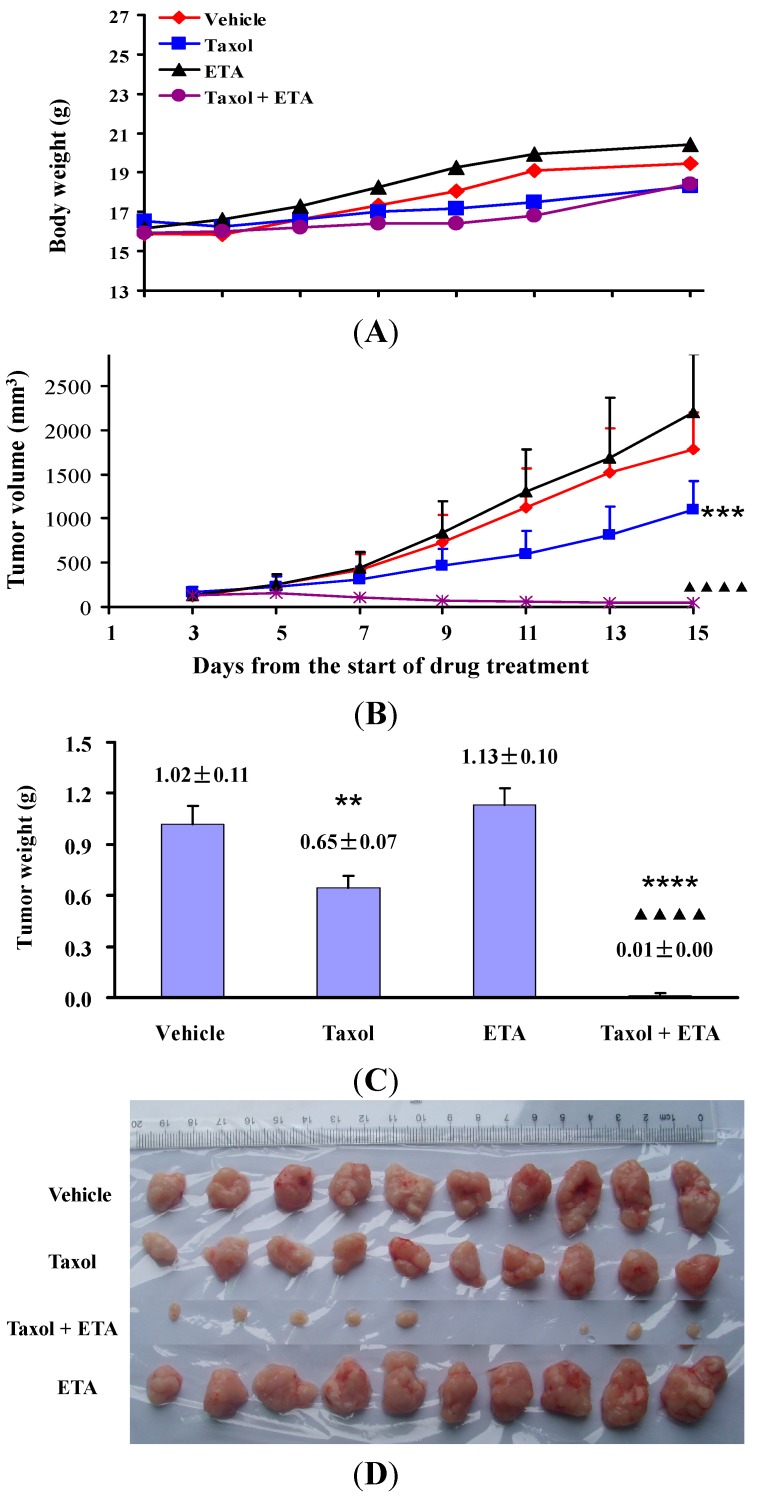
ETA enhanced inhibitory effect of Taxol on growth of KB-3-1 tumor. KB-3-1 tumor-bearing nude mice were given 10 mg/kg Taxol (Taxol) every other day, or 250 mg/kg/day ETA (ETA), or the combination of Taxol and ETA (Taxol + ETA), or equivalent amount of vehicle (Vehicle) for 9 days. Body weight (**A**), tumor volume (**B**) and tumor weight (**C**) were recorded and expressed as mean value ± standard error of mean (n = 10). Tumor picture was taken at the last experimental day (**D**). ** *p* < 0.01, *** *p* < 0.001 and **** *p* < 10^−6^ compared to Vehicle group, while ^▲▲▲▲^ *p* < 10^−6^ compared to Taxol group.

**Figure 2:**
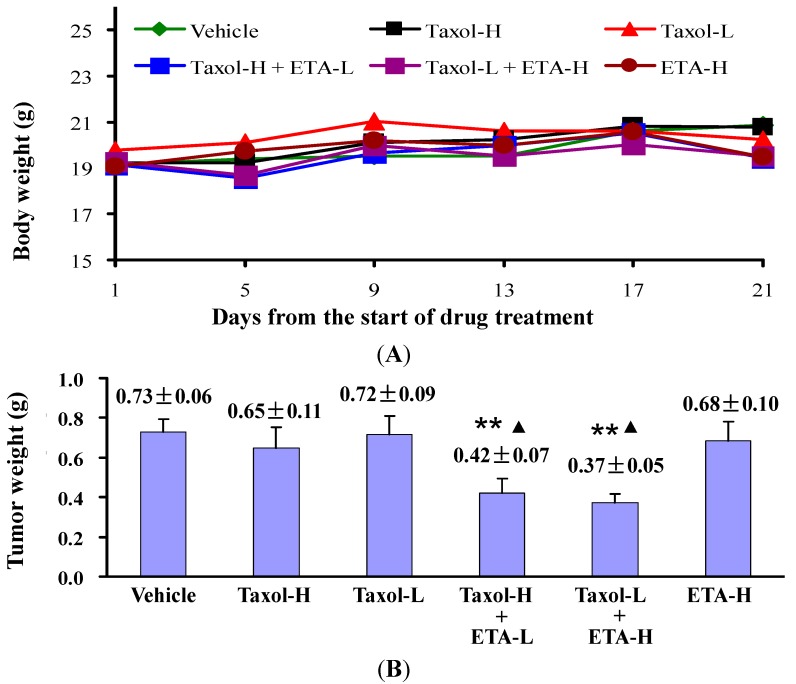
ETA enhanced inhibitory effect of Taxol on growth of HeLa tumors. HeLa tumor-bearing nude mice were given 10 mg/kg (Taxol-H) or 5 mg/kg (Taxol-L) of Taxol every other day for 5 doses, or 200 mg/kg/day of ETA (ETA-H) for 9 doses, or the combination of Taxol-H and 50 mg/kg/day of ETA (Taxol-H + ETA-L), or the combination of Taxol-L and ETA-H (Taxol-L + ETA-H). Body weight (**A**) and tumor weight (**B**) were recorded and expressed as mean value ± standard error of mean (n = 6). Compared to Vehicle group, data were significantly different at ** *p* < 0.01. Compared to Taxol-H group, data were significantly different at ^▲^ *p* < 0.05.

In our study, ETA actively increased the *in vitro* anticancer activity of paclitaxel and was therefore further investigated for its effects on the *in vivo* antitumor ability of paclitaxel. Nude mice bearing HeLa or KB-3-1 xenografts on their back were either given paclitaxel (Taxol) or ETA alone, or their combination. Figure 1 showed the experimental results obtained in KB-3-1 tumor bearing mice. Compared to mice treated with vehicle (vehicle group), oral administration of 250 mg/kg/day of ETA for 9 days (ETA group) did not affect the body weight (Figure 1A), tumor growth trend **(**Figure 1B) and tumor weight (Figure 1C) of mice (*p* > 0.05); treatment with 10 mg/kg Taxol by intraperitoneal injection every other day for five doses (Taxol group) resulted in smaller tumor weight (*p* < 0.001, Figure 1C) and tumor size (Figure 1D). More inspiring efficacy was observed in mice treated with a combination of Taxol and ETA: tumors completely stopped growing and even disappeared in part of the mice (Figure 1D). Compared to treatment with Taxol alone, treatment with Taxol in combination with ETA did not affect the body weight (Figure 1A), but remarkably reduced the average weight and size of tumors (*p* < 10^−6^, Figure 1B‒D), implying that ETA significantly enhanced *in vivo* antitumor activity of paclitaxel. The chemosensitizing effect of ETA was also observed in mice bearing HeLa xenografts. Compared to vehicle, 10 mg/kg of Taxol alone (Taxol-H), or 200 mg/kg of ETA alone (ETA-H) could not inhibit tumor growth, but the combination of 10 mg/kg of Taxol and 50 mg/kg of ETA (Taxol-H + ETA-L), or the combination of 5 mg/kg of Taxol and 200 mg/kg of ETA (Taxol-L + ETA-H) significantly inhibited tumor growth without affecting the body weight of mice (Figure 2A). Tumor weights in the two groups were much lighter than those in the other four groups (Figure 2B).

In tumor therapy, intrinsic or acquired drug resistance in tumor tissue weakens the effect of chemotherapy treatment, and more seriously, can result in treatment failure. The mechanisms of drug resistance involved include over-expression of transporter proteins such as Pgp, MRP or BCRP, over-expression of glutathione S-transferease, mutation of tumor suppressor gene p53, up-regulation of topoisomerase II or topoisomerase II gene mutation, *etc.* [2]. Paclitaxel is an effective drug for gynecological tumors, including breast cancer, ovarian cancer, cervical carcinoma, and carcinoma of the endometrium. Paclitaxel is also a transport substrate of Pgp, an ATP-dependent membrane transporter protein that pumps substrate drug out of tumor cells [19]. The HeLa tumor model established by subcutaneously injecting HeLa cells on the back of nude mice was reported to be highly sensitive to paclitaxel [20], but HeLa tumors in this study grew well, even when exposed to 10 mg/kg of paclitaxel, implying that *in vivo* HeLa tumor model established in this experiment was resistant to paclitaxel. In our study, paclitaxel or ETA alone could not inhibit tumor growth, but paclitaxel in combination with ETA significantly inhibited tumor growth, meaning that the paclitaxel sensitivity of the resistant HeLa tumor was restored by ETA.

The incidence of oral cancer is high in areas where betel nut and products derived from it are socially endorsed masticatory products [21]. In our study, treatment with 10 mg/kg of paclitaxel alone significantly slowed down the growth of KB-3-1 tumors, but could not stop the tumor growth. However, when paclitaxel was given together with ETA, the growth of KB-3-1 tumors was completely inhibited.

In this study, co-administration of paclitaxel with ETA achieved much better therapeutic effect than administration of paclitaxel alone, indicating that ETA is a potential paclitaxel sensitizer in treatment of human cancer. Considering that paclitaxel is a transport substrate of Pgp while tenacigenin B derivatives could inhibit Pgp transport function [17], it is strongly suggested that ETA might work through inhibiting Pgp function.

### 2.3. Chemical Composition of ETA

**Figure 3:**
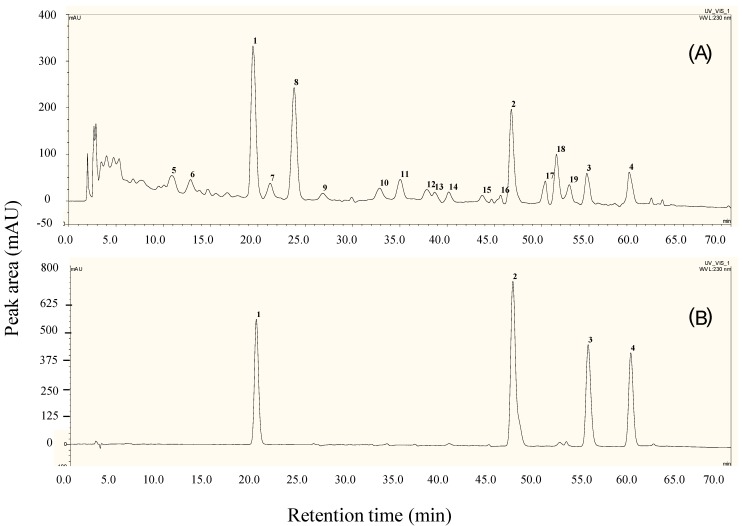
Characteristic chromatograms of ETA and the markers assayed by HPLC. (**A**) ETA; 1–19, detectable components; (**B)** the mixed markers, 1–4, marker compounds **1**–**4**. For assay, methanol solutions of mixed markers **1**–**4** (1.04, 1.21, 0.98, 0.96 mg/mL) and of ETA (1.42 mg/mL) were used, oven temperature was set at 30 °C, and detecting wavelength was set at 230 nm.

Chemical constituents of ETA were well separated through a Phenomenex Luna C_18_ column (4.6 × 250 mm, 5 μm) eluting with methanol (solvent A) and 0.1% aqueous acetic acid (*v*/*v*, solvent B), at oven temperature 30 °C and detection wavelength 230 nm with gradient elution: 0–30 min, 58%–63% A; 30–40min, 63%–68% A; 40–70 min, 68%–78% A. The HPLC fingerprints of ETA are shown in Figure 3A. Chromatograms of this active extract were characterized by the presence of a group of tenacigenin B ester derivatives, including 11α-*O*-tigloyl-12β-*O*-acetyltenacigenin B (**1**), 11α,12β-di-*O*-tigloyltenacigenin B (**2**), 11α-*O*-2-methylbutanoyl-12β-*O*-tigloyltenacigenin B (**3**), and 11α-*O*-(2-methylbutanoyl)-12β-*O*-benzoyltenacigenin B (**4**) (Figure 3B), which were also numbered as peaks 1–4 in the chromatogram. According to the sum of peak area, marker compounds **1**–**4** accounted for 42.14% content of that of 19 detectable components in ETA (Supplementary materials; Table S1).

### 2.4. Assay of **1**

Reference compound **1** was shown to be a major constituent of ETA (Figure 3) and was therefore measured quantitatively. The condition optimized for assay of **1** was the same as described in compositional analysis of ETA, except that isocratic elution using methanol mixed with 0.1% aqueous acetic acid (65:35; *v*/*v*) was chosen. The range of relative standard deviations for accuracy of peak area, repeatability of measured concentration, and stability of sample within 12 h were validated as 0.42%–0.79%. The calibration curve for **1** was Y = 264.04X + 2.0137 (R^2^ = 0.9998) with a linear range of 1.04–16.64 μg. Recovery rates for spiked samples of ETA were determined as 100.65% ± 1.03%. Measured concentration of **1** was 11.88% ± 0.50% (n = 3).

## 3. Experimental Section

### 3.1. Preparation of the Extract of Total Aglycones of M. tenacissima

The plant was collected from Mengzi County, Yunnan Province, China in October 2011 and identified as *Marsdenia tenacissima* (Roxb.) Wight et Arn. by Fa-Guo Wang, a plant taxonomist at South China Botanical Garden, Chinese Academy of Sciences. A voucher specimen (No. 201110 sp-2) was deposited at the Chinese Herbal Drug Discovery Lab of theTropical Medicine Institute, Guangzhou University of Chinese Medicine. Dried and ground stems of *M. tenacissima* (8.0 kg) were extracted three times with 80% (*v*/*v*) ethanol (50L) under reflux, 2 h each time. The combined ethanol solution was concentrated *in vacuo* to remove ethanol and extracted successively with petroleum ether (PE), ethyl acetate (EtOAc) and *n*-butanol (BuOH). After removal of organic solvents and drying *in vacuo*, PE, EtOAc, and BuOH extracts were obtained. EtOAc extract (80.0 g) showed positive Lieberman-Burchard (L-B) and Keller-Kiliani (K-K) reactions, suggesting the concentration of steroidal glycosides containing 2-deoxyhexose residues and was named as extract of total glycosides [22,23]. A sample of the total glycosides (40 g) was dissolved in anhydrous EtOH (500 mL), mixed with 0.05 M H_2_SO_4_ (500 mL) and refluxed for 1 h. The reactive mixture was neutralized by 10% Na_2_CO_3_, distilled *in vacuo* to remove EtOH, and extracted with EtOAc. The EtOAc extract gave a positive L-B reaction and negative K-K reaction, suggesting the concentration of total aglycones [22,23], and was named as ETA (22 g).

### 3.2. In Vitro Anticancer Activity of Paclitaxel in the Presence or Absence of ETA

Human hepatoma cell line HepG2 and human leukemia cell line K562 were purchased from the cell bank of the Chinese Academy of Science (Shanghai, China). Human oral epidermoid carcinoma cell line KB-3-1 and human cervical cancer cell line HeLa were kindly provided by Dr. Tse AK of Hong Kong Baptist University. K562, HeLa and HepG2 cell lines were maintained in RPMI 1640 (Gibco-BRL, Grand Island, NY, USA) containing 10% fetal bovine serum (Gibco-BRL), while KB-3-1 cell line was maintained in DMEM (Gibco-BRL) supplemented with 10% fetal bovine serum. All cells were cultured at 37 °C, saturated humidity and 5% CO_2_. To examine the inhibitory effect of paclitaxel alone or in combination with ETA on cancer cell viability, 100 μL per well of cell suspension containing 1 × 10^4^ cells was seeded into a 96-well microplate and incubated 24 h. Cells were then exposed to various concentrations of paclitaxel (Sigma/Aldrich Co., St Louis, MO, USA) in the presence or absence of ETA (10 μg/mL) for 48 h. Cell viability was then measured using Cell Counting Kit-8 (Dojindo Laboratories, Shanghai, China) [24]. IC_50_ values of paclitaxel in the presence and absence of ETA were used to evaluate the *in vitro* anticancer activity.

### 3.3. In Vivo Anti-Tumor Effect of Paclitaxel in the Presence or Absence of ETA

Animal studies were approved by the Animal Ethics Committee at Guangzhou University of Chinese Medicine (Document No. syxk (Yue) 2008-0001) and performed according to its Animal Care and Use Guidelines. Female BALB/c nude mice of 4–5 weeks of age were subcutaneously injected with 5 × 10^6^ of HeLa cells or 3 × 10^6^ of KB-3-1 cells on the back to establish tumor xenografts [25,26]. Two days after KB-3-1 cell injection, mice were randomly divided into four groups (10 mice each group) and treated with 10 mg/kg of paclitaxel by intraperitoneal injection (Taxol group), or 250 mg/kg of ETA by oral administration (ETA group), or the combination of paclitaxel and ETA (Taxol + ETA group). Mice given equivalent amount of solvent (8.6% of Cremophor EL, 8% of ethanol in saline, 10 mL/kg by intraperitoneal injection) were set as drug negative control (Vehicle group). Drug treatment lasted for 9 days. Paclitaxel was given every other day for five doses while ETA was given orally daily. Body weight and tumor size were recorded every other day. Tumor size was monitored by measuring two perpendicular diameters with a caliper and tumor volume was calculated as volume = length × width^2^ × 0.5. The animal experiments were terminated 6 days after the last drug treatment by sacrificing mice according to the guidelines. Tumor xenografts were then stripped and weighed.

Twelve days after injection of HeLa cells, mice with tumors growing well were selected and randomly divided into four groups (six mice each group) and treated with 10 mg/kg (Taxol-H group) or 5 mg/kg (Taxol-L group) of paclitaxel, or 200 mg/kg of ETA (ETA-H group), or the combination of 10 mg/kg of paclitaxel and 50 mg/kg of ETA (Taxol-H + ETA-L), or the combination of 5 mg/kg of paclitaxel and 200 mg/kg of ETA (Taxol-L + ETA-H group), or equivalent volume of solvent (Vehicle group). Drug treatment lasted for 9 days and pacltaxel and ETA were given as described above. Body weights of mice were monitored routinely. The experiment was terminated 12 days after the last drug treatment and the tumor xenografts were obtained.

### 3.4. Statistical Analysis

Data were expressed as Mean ± standard error of the mean (SEM). Effects of various treatments were analyzed by ONE-WAY ANOVA analysis. *p* value < 0.05 was considered statistically significant.

### 3.5. Chemical Compositional Analysis of ETA

Chemical composition of ETA was assayed by reversed phase HPLC and characterized by the presences of a group of tenacigenin B esters as marker of quality control. The reference compounds used in qualitative analysis were 11α-*O*-tigloyl-12β-*O*-acetyltenacigenin B (**1**) [27], 11α,12β-di-*O*-tigloyltenacigenin B (**2**) [28], 11α-*O*-2-methylbutanoyl-12β-*O*-tigloyltenacigenin B (**3**) [27], and 11α-*O*-(2-methylbutanoyl)-12β-*O*-benzoyltenacigenin B (**4**) [27]. These compounds were isolated from ETA by chromatography, and their structures were identified by analysis and comparison on their NMR and MS data with those reported. NMR (Tables S2, S3) and MS data of **1**–**4** were listed in the Supplementary materials. Chromatographic conditions involving C-18 column, mobile phase, and elution manner were screened and optimized.

### 3.6. Assay of Marker Compound **1**

Compound **1** was used as assay marker. Mobile phase, standard curve, linearity of calibration curve, precision, stability, reproducibility, and accuracy were verified.

## 4. Conclusions

This is the first demonstration that total pregnane aglycones (ETA) manufactured from the plant *M. tenacissima* markedly enhanced the anticancer activity of paclitaxel both *in vitro* and *in vivo*. Our data support the use of ETA as a sensitizer for paclitaxel treatment of cancer. Results of HPLC assays on ETA provided a basis of quality control for this chemosensitizer.

## Supplementary Materials

Supplementary materials can be accessed at: http://www.mdpi.com/1420-3049/19/9/13965/s1.

## Supplementary Files

Supplementary File 1
