# Supplementary material for: Total Aglycones from Marsdenia tenacissima Increases Antitumor Efficacy of Paclitaxel in Nude Mice
Source: Molecules. 2014 Sep 5;19(9):13965–75. doi: 10.3390/molecules190913965 (PMC6271049; doi:10.3390/molecules190913965)
Supplement: Supplementary File 1 [file molecules-19-13965-s001.pdf]

Supplementary Materials

1: 11α-*O*-Tigloyl-12β-*O*-acetyl-tenacigenin B ($C_{28}H_{30}O_7$); ESI-MS: *m/z* 511.5 ($[M+Na]^+$)

2: 11α,12β-di-*O*-Tigloyl-tenacigenin B ($C_{31}H_{44}O_7$); ESI-MS: *m/z* 551.4 ($[M+Na]^+$)

3: 11α-*O*-2-Methylbutanoyl-12β-*O*-tigloyl-tenacigenin B ($C_{31}H_{46}O_7$); ESI-MS: *m/z* 553.4 ($[M+Na]^+$)

4: 11α-*O*-2-Methylbutanoyl-12β-*O*-benzoyl-tenacigenin B ($C_{33}H_{44}O_7$); ESI-MS: *m/z* 575.3 ($[M+Na]^+$)

Table S1. Peak area of detectable component in ETA (mAU). HPLC condition: Phenomenex Luna C_{18} column (4.6 × 250 mm, 5 μm); oven temperature: 30 °C; detecting wavelength: 230 nm; mobile phase: methanol (A) and 0.1% aqueous acetic acid (*v*/*v*, B); gradient elution: 0–30 min, 58%–63% A; 30–40 min, 63%–68% A; 40–70 min, 68%–78% A.

No. of Peak	Peak Area (mAU)	Area %
1 (**1**)	196.21	20.09
2 (**2**)	119.47	12.23
3 (**3**)	40.31	4.13
4 (**4**)	55.58	5.69
5	62.07	6.35
6	36.35	3.72
7	24.43	2.50
8	158.20	16.20
9	11.44	1.17
10	35.71	3.66
11	44.52	4.56
12	24.12	2.47
13	14.23	1.46
14	14.07	1.44
15	10.05	1.03
16	8.52	0.87
17	27.56	2.82
18	61.86	6.33
19	32.06	3.28
Sum	976.77	100

Table S2. ^{13}C-NMR data for compounds **1–4** in $CDCl_3$ (100 MHz, δ: ppm).

C	1	2	3	4
1	37.1	37.2	37.5	37.5
2	31.2	31.4	31.1	31.0
3	70.4	70.6	70.4	70.3
4	38.2	38.4	38.2	38.2
5	43.9	44.0	44.0	44.0
6	26.5	26.7	26.6	26.6
7	31.7	31.8	31.7	31.7
8	66.8	66.9	66.8	66.9
9	51.1	51.2	51.0	51.0
10	38.8	38.8	38.9	38.9
11	68.6	68.8	68.5	68.4
12	75.0	74.7	74.6	75.3
13	45.7	46.0	46.0	46.0
14	71.3	71.5	71.4	71.4
15	26.5	26.7	26.5	26.5
16	24.9	25.0	24.9	24.9
17	59.7	59.8	59.9	59.9
18	16.5	16.6	16.7	16.7
19	12.6	12.7	12.7	12.8
20	210.8	210.9	210.8	210.8
21	30.0	30.2	29.9	29.9
Acyl1	Tig	Tig	Bu	Bu
1	167.2	167.3	175.7	175.7
2	128.5	127.9	41.3	41.1
3	138.0	137.7	25.8	25.7
4	14.4	14.3	11.6	11.4
5	11.9	11.7	15.1	15.0
Acyl2	Ac	Tig	Tig	Bz
1	170.7	167.4	167.3	166.0
2	20.5	128.7	128.0	129.4
3		138.0	138.5	129.8
4		14.3	14.4	128.4
5		11.8	11.8	133.2
6				128.4
7				129.8

Table S3. ^{1}H-NMR data for compound **1–4** in $CDCl_3$ (400 MHz; δ: ppm; *J*: Hz).

**H**	**1**	**2**	**3**	**4**
C-3	3.556 (1H, m)	3.559 (1H, m)	3.574 (1H, m)	3.587 (1H, m)
C-9	2.008 (1H, d, 10.0)	2.018 (1H, d, 10.4)	2.003 (1H, d, 10.4)	2.065 (1H, d, 10.4)
C-11	5.393 (1H, t, 10.0)	5.449 (1H, t, 10.0)	5.389 (1H, t, 10.0)	5.523 (1H, t, 10.0)
C-12	4.992 (1H, d, 10.0)	5.032 (1H, d, 10.4)	5.023 (1H, d, 10.0)	5.222 (1H, d, 10.0)
C-17	2.907 (1H, d, 7.6)	2.907 (1H, d, 7.2)	2.897 (1H, d, 7.2)	2.962 (1H, d, 7.2)
C-18	1.082 (3H, s)	1.091 (3H, s)	1.063 (3H, s)	1.139 (3H, s)
C-19	1.045 (3H, s)	1.052 (3H, s)	1.040 (3H, s)	1.074 (3H, s)
C-21	2.171 (3H, s)	2.185 (3H, s)	2.201 (3H, s)	2.253 (3H, s)
Acyl1	Tig	Tig	Bu	Bu
C-4′	1.732 (3H, brs)	1.683 (3H, d, 7.2)	0.773 (3H, t, 7.6)	0.535 (3H, t, 7.6)
C-5′	1.747 (3H, s)	1.729 (3H, s)	0.958 (3H, d, 7.2)	0.836 (3H, d, 7.2)
Acyl2	Ac	Tig	Tig	Bz
C-2″	1.834 (3H, s)			
C-3″				7.937 (1H, d, 7.2)
C-4″		1.683(3H, d, 7.2)	1.735 (3H, d, 5.6)	7.369 (1H, t, 7.6)
C-5″		1.729 (3H, s)		7.532 (1H, t, 7.6)
C-6″				7.369 (1H, t, 7.6)
C-7″				7.937 (1H, d, 7.2)
